# Experimental and Computational Comparison of Intervertebral Disc Bulge for Specimen-Specific Model Evaluation Based on Imaging

**DOI:** 10.3389/fbioe.2021.661469

**Published:** 2021-05-28

**Authors:** Marlène Mengoni, Fernando Y. Zapata-Cornelio, Vithanage N. Wijayathunga, Ruth K. Wilcox

**Affiliations:** School of Mechanical Engineering, Institute of Medical and Biological Engineering, University of Leeds, Leeds, United Kingdom

**Keywords:** intervertebral disc, bulge, modelling, reverse engineering, MRI

## Abstract

Finite element modelling of the spinal unit is a promising preclinical tool to assess the biomechanical outcome of emerging interventions. Currently, most models are calibrated and validated against range of motion and rarely directly against soft-tissue deformation. The aim of this contribution was to develop an *in vitro* methodology to measure disc bulge and assess the ability of different specimen-specific modelling approaches to predict disc bulge. Bovine bone-disc-bone sections (*N* = 6) were prepared with 40 glass markers on the intervertebral disc surface. These were initially magnetic resonance (MR)-imaged and then sequentially imaged using peripheral-qCT under axial compression of 1 mm increments. Specimen-specific finite-element models were developed from the CT data, using three different methods to represent the nucleus pulposus geometry with and without complementary use of the MR images. Both calibrated specimen-specific and averaged compressive material properties for the disc tissues were investigated. A successful methodology was developed to quantify the disc bulge *in vitro*, enabling observation of surface displacement on qCT. From the finite element model results, no clear advantage was found in using geometrical information from the MR images in terms of the models’ ability to predict stiffness or disc bulge for bovine intervertebral disc.

## Introduction

Globally, back pain causes more disability than any other condition (James et al., 2018). While the specific causes are often unclear, changes to the structure and morphology of the intervertebral disc (IVD) are frequently implicated (de Schepper et al., 2010; Brinjikji et al., 2015). The development of new surgical interventions for the IVD have been hampered by the limitations in current preclinical testing methods. In particular, *in vitro* testing is challenging due to the hydrated nature of the tissues and natural variation that occurs between samples (Vadalà et al., 2015; Sikora et al., 2018). *In silico* finite element (FE) models are a promising preclinical testing tool, capable of targeting specific situations, organ/tissue behaviour, and accounting for population variation (Schmidt et al., 2007; Mengoni et al., 2016). In particular, there is potential for FE analysis to be used to examine nucleus augmentation or replacement where design variables, such as the biomaterial properties or device size, and patient variables such as the properties of the surrounding tissues can be parametrically altered and evaluated. The credibility of such models depends, among other things, on the correct description of the mechanical properties for a specific context of use and on relevant validation (Jones and Wilcox, 2008).

Both calibration and validation of *in silico* models of the spine have been commonly undertaken using averaged data from *in vitro* experiments, including range of motion and load-displacement behaviour as well as IVD pressure (e.g., Schmidt et al., 2006, 2007; Ayturk and Puttlitz, 2011; Adam et al., 2015; Brummund et al., 2017). Since there often exists large specimen-to-specimen variation in the measured values, this approach does not allow direct validation or calibration of an individual specimen behaviour.

Comparisons between specimen-specific models and corresponding *in vitro* experimental tests of the same specimen have been undertaken in a limited number of studies, using global measures of behaviour such as stiffness or range of motion (e.g., Maquer et al., 2015; Sikora et al., 2018; Stadelmann et al., 2018). A number of methods of measuring localised deformation or strain have been applied to the disc, such as laser scanning (Heuer et al., 2007; Fewster et al., 2020), potentiometer based surface tracking (Brinckmann and Horst, 1985; Brinckmann and Grootenboer, 1991), stereo digital image correlation (Spera et al., 2011), and magnetic resonance (MR) imaging (O’Connell et al., 2007, 2011; Showalter et al., 2016; Tavana et al., 2020). However, these methods of measuring local tissue displacement have not yet been applied to validate specimen-specific *in silico* models. Local tissue displacement such as disc bulge can be a good measure to evaluate the capacity of a model to replicate the biomechanics of the disc directly, rather than of the disc and the motion segment geometry analysed through global measures. Disc bulge is also an indirect measure of the capacity of the annulus to sustain the nucleus compression.

Specimen-specific FE models are often constructed from computed tomography (CT) or MR image data. The use of high-resolution CT image data is considered to be the gold standard for the development of computational models of hard tissues due to the feasibility of generating image-based material parameters and the reliability of the image data (Jones and Wilcox, 2008). However, a CT protocol optimised for hard tissues will not be able to effectively provide information on IVD soft tissue structures. Magnetic resonance imaging can allow different soft tissue structures to be differentiated, although limitations of resolution in 3D scans, coupled with sensitivities to tissue condition and orientation (Wijayathunga et al., 2019), make the use of MR data more challenging for model generation.

Since both CT and MR data can be acquired in preclinical studies, there is potential to use both modalities to combine the advantages of each. When testing tissue *ex vivo* for preclinical assessment of therapies, it is not clear which geometrical information from both modalities affects the accuracy of FE models of the IVD for information of interest such as stiffness or bulge. Therefore, the main aims of this study were:

1. To develop a methodology to quantify the local surface deformations of the IVD *in vitro* (“disc bulge”) in such a way that direct comparisons could be made with FE model predictions.
2. To assess the ability of a simple specimen-specific finite element (FE) methodology to predict the disc bulge, using different approaches to represent the internal geometry of the IVD.

## Materials and Methods

**FIGURE 1:**
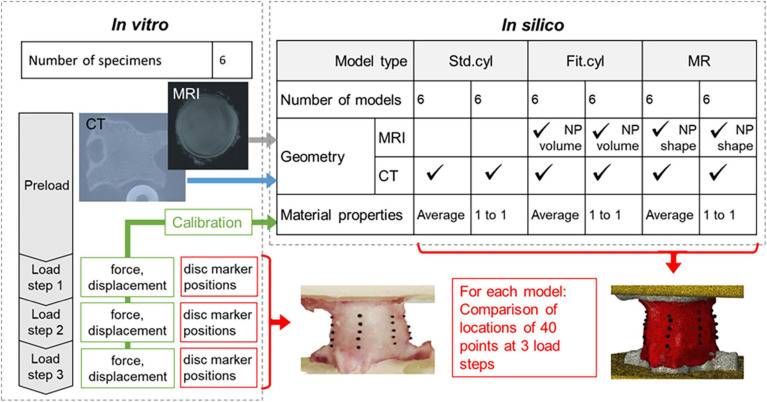
Overview of the experimental *in vitro* and computational *in silico* arms of the study.

Bovine caudal IVDs were used in this study due to the similarities to human discs (Beckstein et al., 2008) and the lack of facet joints; this tissue is also often used in preclinical assessment of intervertebral disc repair (e.g., Chan et al., 2010; Miles et al., 2016; Hom et al., 2019; among other groups). The tissue was obtained from food-chain animals for which no ethical approval was required. In the experimental arm of the study, the IVDs were imaged using MRI before being sequentially loaded and imaged under CT. In the computational arm, FE models were generated from the initial image data. Experimental load-displacement values were used to calibrate the compressibility of the disc tissues, whilst the disc surface bulge, measured experimentally at 40 points, was used for point-wise comparison of the predicted disc displacement. The experimental and computational arms of this study are described in detail below and summarised in Figure 1.

### Specimen Preparation, Imaging, and Mechanical Testing

**FIGURE 2:**
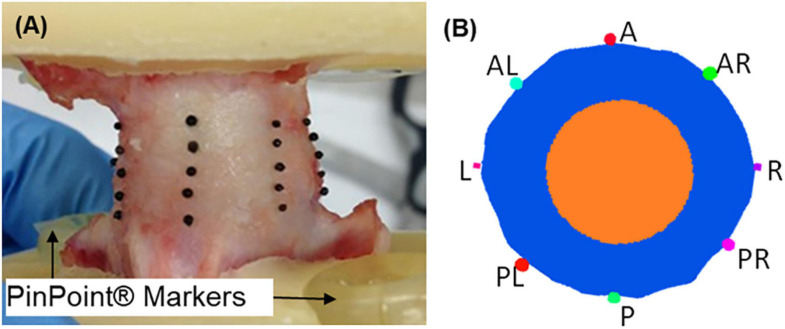
**(A)** Osteodisc prepared for mechanical testing, with glass fiducial markers, CT- and MR-compatible PinPoint^®^ markers, and PMMA endcaps. **(B)** Transverse plane slice through the intervertebral disc showing segmentation of the annulus fibrosus (blue), nucleus pulpous (orange) and fiducial glass beads, with classification of the eight zones of the disc (A, anterior; P, posterior; L, left; R, right).

The bovine tissue was sourced from a local abattoir and frozen at −80°C prior to use. Six osteodiscs (half vertebra-disc-half vertebra sections) were extracted from coccygeal levels 1–4 of two tails by making parallel cuts through adjacent vertebrae, leaving approximately 15 mm of bone on each side of the IVD (Sikora et al., 2018). The specimens were potted into polymethyl methacrylate (PMMA) endcaps (Figure 2A). CT- and MR-compatible large markers (PinPoint^®^ Multi-Modality Fiducial Marker, Oncology Imaging Systems Ltd., United Kingdom) were embedded into the surface of the endcaps. Additionally, forty glass fiducial markers (∼1 mm diameter) were attached to the surface of each disc using petroleum jelly where required. These were arranged along eight locations on craniocaudal lines spaced at 45-degree intervals (Figure 2B), and with five glass markers per line.

Each potted osteodisc was placed between parallel Delrin fixtures in a rig that facilitated consistent positioning and alignment relative to the global axes on a 3T MR platform (MAGNETOM^®^ Verio, Siemens Healthineers, Erlangen, Germany). The specimens were imaged using a T2-weighted turbo spin-echo sequence with 0.3 × 0.3 mm in-plane resolution and slice thickness of 1 mm.

**FIGURE 3:**
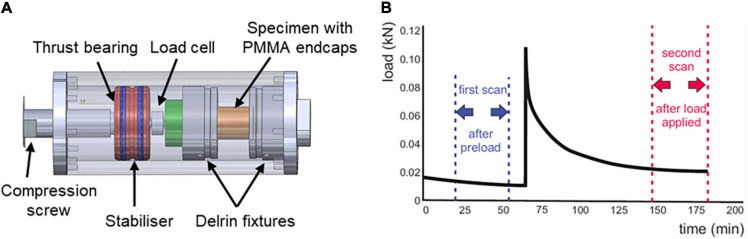
**(A)** The CT-compatible compression rig. **(B)** Illustration of the timing of the CT image acquisition, showing the first “preloaded” scan and second scan following load step 1.

The specimens were transferred to a CT-compatible compression rig (Figure 3A) fabricated in-house (Sikora, 2013). The rig enabled the specimens to be manually compressed via a screw-driven piston with transverse displacements and rotations constrained; the applied load was measured using a universal load cell (SLC31/01000, RDP Electronics Ltd., United Kingdom).

Each specimen was then axially compressed and imaged in a high-resolution peripheral-qCT scanner (XtremeCT, Scanco Medical, Switzerland) at an isotropic resolution of 82 μm (60 kVp and 900 μA), under a constant displacement. The specimen was placed between parallel platens and an initial pre-load of 50 N was applied to maintain alignment prior to the first scan. This was followed by four sequential axial compression displacement steps of approximately 1 mm each with a hold period of 90 min after each step prior to the subsequent CT scan to allow the specimen to relax (Figure 3B). This displacement increment was selected following pilot studies to enable the disc marker lateral displacements to be measurable at the image resolution. The exact applied displacement for each step was measured from the image data during image processing. The manual rotation of the screw to apply the displacement was performed very slowly (>30 s) to keep the strain-rate low.

### Image Processing and *in vitro* Bulge Calculation

Computed tomography images were imported into Simpleware ScanIP (v 7.0, Synopsys, United States) after their greyscale-values had been rescaled to enable the use of previously calibrated bone properties (Zapata-Cornelio et al., 2017). The rescaled CT images at each compression step were rigidly registered to the CT images of specimens in their initial preloaded state (“preloaded CT images”), using the most caudal vertebrae as reference. Coordinates for all glass markers on the registered images were automatically calculated, using image processing and recognition (Python v2.7 with OpenCV v3.0). This automatic method was validated by manually extracting the marker positions on the CT images for one specimen.

Intervertebral disc bulge at each marker was calculated as the displacement of the marker centroid in the transverse plane, between the deformed and the initial stage.

Magnetic resonance images were resampled to the same resolution as the CT images, and rigidly registered to the preloaded CT images, using the PinPoint^®^ markers as references.

### Finite Element Modelling

**FIGURE 4:**
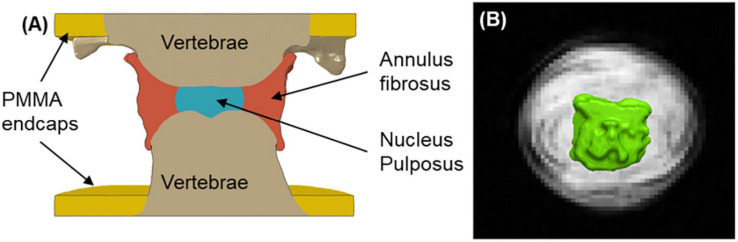
**(A)** Longitudinal section through an FE model of one specimen (std. cyl. model). **(B)** Volume rendering (in green) of an MRI-segmented nucleus pulposus, with an axial slice through the centre of the disc, showing defined annulus fibrosus lamellar structure.

The preloaded CT images were segmented in Simpleware ScanIP to separate bone, intervertebral disc, individual glass markers, and cement endcaps (Figure 4A), using automatic thresholding and manual operations for the outer surface of the annulus fibrosus (AF). The segmented markers were used to identify the closest node on the disc surface and were not subsequently included in the FE analysis. The registered MR images were segmented to isolate the nucleus pulposus (NP) and compute its volume (Figure 4B).

Given that accurate distinction of different soft tissue structures using image contrast was difficult on the CT scan, the NP and AF were separated as below to create three nucleus model types:

1. A cylindrical NP, centred within the AF in the transverse plane, with dimensions in such a way that NP:AF diameter ratio was 0.5 (Mengoni et al., 2017) (**std. cyl. model**);
2. A cylindrical NP, centred within the AF in the transverse plane, with dimensions so that the volume of the NP matched that measured on the MR image of the specimen (**fit. cyl. model**);
3. The NP geometry directly derived from the segmented MR image (**MR model**).

All specimens were modelled in a specimen-specific approach, with non-linear quasi-static FE analysis run in parallel using Abaqus CAE 2017 (Simulia, Dassault Systèmes).

The 18 models (six specimens each with three nucleus model types) were meshed with linear tetrahedral elements with size between 0.5 and 1.0 mm following previous mesh convergence studies (Jones and Wilcox, 2007; Luxmoore, 2013, see data associated with this paper for mesh size comparison). This led to an average of 1.15 million elements per model (range 0.9–1.6 million), 620 thousand of which represented the IVD (range 400–850 thousand). Boundary conditions were applied to simulate the experimental setup: the inferior face of the lower endcap was clamped while the superior face of the upper endcap was constrained in translation and rotation in the transverse plane. An axial compressive displacement was applied to the upper endcap, the magnitude of which was matched to the displacement measured in successive CT scans. The constitutive model and parameters used for the AF have been validated for a compression up to 30% (Sikora et al., 2018). As it is not known if it is valid for the displacement obtained in the fourth experimental step which yield over 50% compression, only the first three experimental compression steps were modelled.

The material parameters are summarised in Table 1. Bone was modelled as a linear elastic material, with element-by-element elastic modulus scaled using the underlying greyscale value of the CT image data, and Poisson’s ratio of 0.3. In order to use a greyscale-based equation for the bone modulus (Zapata-Cornelio et al., 2017), the images underlying the high-resolution segmentation were down-sampled to an isotropic resolution of 0.5 mm. The greyscale-based model requires first a normalisation of the greyscale to 0–255. A linear mapping between the greyscale and the element elasticity modulus is then applied, with a mapping coefficient previously validated for bovine tails (Zapata-Cornelio et al., 2017). The cement endcaps were assigned an elastic modulus of 2.45 GPa, and Poisson’s ratio of 0.3 (Tarsuslugil et al., 2014). The annulus fibrosus was modelled using a Holzapfel exponential model (Gasser et al., 2006), with an oblique/counter-oblique fibre orientation at 20° to the transverse plane and fibre properties previously validated for a similar axial loading setup (Mengoni et al., 2017; Sikora et al., 2018). The nucleus pulposus was modelled as a Mooney-Rivlin material (Adam et al., 2015).

**TABLE 1:** Material parameters.

Linear elastic materials					
	E (MPa)		*v* (–)		
Bone	Linear dependency with greyscale, ranging from 3.6 MPa to 842 MPa (median 386.8 MPa)		0.3		Zapata-Cornelio et al., 2017
PMMA Cement	1035		0.3		Tarsuslugil et al., 2014

**Hyperelastic materials**					

	K (MPa)	C_10_ (MPa)	k_1_ (MPa)	k_2_ (–)	
Annulus (G.O.H. model)	Calibrated for load	0.25	1.43	1.63	Mengoni et al., 2017
	K (MPa)		C_10_ (MPa)	C_01_ (MPa)	
Nucleus (Mooney-Rivlin model)	Calibrated for load		0.07	0.02	Adam et al., 2015

For each model, NP and AF compressibility values were calibrated in order to replicate the experimental peak axial load achieved at each displacement step (**1-to-1 calibration**), producing different values of tissues compressibility for each model. The cost function of the optimisation was the RMS difference between the experimental and computational loads for each displacement step, optimised using a Trust Region Reflective method with opti4Abq (Mengoni, 2021). The calibration process was considered successful when the cost function was below 10% of the maximum experimental load, with initial conditions using the compressibility of water (1/2,200 MPa^–1^) for both AF and NP. Only the compressibility values were calibrated because they have been shown to have the largest effect on the outcome of this type of models (Mengoni et al., 2017). As a comparison, FE models were also run where the compressibility of the disc tissues was set to the average value across all six specimens of the 1-to-1 calibrations within each nucleus model type (**average compressibility** models). Here the same value of tissue compressibility was applied to all specimens within each nucleus model type.

### Data Analysis

The data associated with this study (CT and MR images, key anatomical measurements, FE models, and results of calibration) is openly available on the University of Leeds data repository (Mengoni and Wilcox, 2019).

All statistical analysis was performed using R v.3.5.0 (R Foundation for Statistical Computing) and statistical significance was set at *p* < 0.05.

#### Experimental Data

The applied displacement was measured from the micro-CT scan, measuring the distance between the inner surfaces of the PMMA endcaps.

To compare bulge values across samples and marker locations at each step, the *in vitro* bulge at each marker was normalised with respect to the applied displacement at each step. The normalisation was required as each specimen had a slightly different applied displacement. The normalised bulge was compared between the eight marker locations with a Welch-corrected ANOVA test after assessing normality with a Shapiro test and heteroscedasticity with a Levene test. Where a statistically significant difference was found, a post-hoc analysis was performed using a pairwise *t*-test with pooled SD and Bonferroni correction.

#### Computing Equivalent Poisson’s Ratio

Compressibility values obtained by the optimisation algorithm were transformed into equivalent Poisson’s ratio, assuming a linear-equivalent material model for both the annulus fibrosus and the nucleus pulposus (for which the shear modulus *G* = 2*C*_10_; Timoshenko and Goodier, 1970):

$$v=\frac{3K-4C_{10}}{6K+4C_{10}}$$

Where *K* is the bulk modulus value obtained from the optimisation of compressibility, *C*_10_ = 0.07*MPa* for the nucleus pulposus (Table 1) and *C*_10_ = 1.92*MPa* for the annulus fibrosus (value from a linear fit at small strain of the stress/strain data obtained from the Holzapfel model and parameters as in Table 1). Such a Poisson’s ratio is provided as an easily interpretable parameter for comparison rather than a material parameter used in a model.

#### Comparison of Experimental and Computational Data

The computational axial load required for each displacement step was compared to the experimental equivalent using concordance correlation coefficients for each model type (three types of nucleus models and either one-to-one calibration or average compressibility). The concordance correlation coefficient is a measure of the agreement between values measured by two methods (Lin, 1989), hence of the ability of the *in silico* models to reproduce *in vitro* data.

As well as comparing bulge values between marker locations similarly to the experimental data analysis, the bulge was compared between *in vitro* and *in silico* values for each model type using a concordance correlation coefficient. Markers for which experimental displacement was in the lower tenth percentile were not included in this analysis to avoid comparisons with the smallest experimental displacements, which are likely to be more error-prone.

## Results

### Experimental Specimens Mechanical Testing

The NP volume computed from the MR images ranged from 7 to 17% of the disc volume, with equivalent NP diameters between 26 and 41% of the corresponding AF average diameters. These volumes were consistently smaller than the standard cylindrical NP used in the std. cyl. FE models, which assumed an NP:AF diameter ratio of 50%.

**FIGURE 5:**
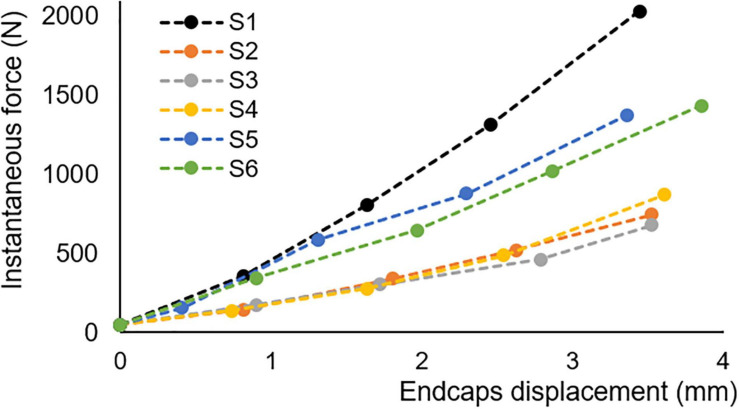
*In vitro* load-displacement for all specimens, extracted from 2 spines (samples S1, S5, and S6 come from one spine; samples S2–4 come from the other spine); the load is the peak load measured, the displacement is as measured on CT scans with a resolution of 82 μm.

The mechanical testing data showed a stiffening behaviour through the four loading steps (Figure 5). The applied displacement at each step across all samples was 0.89 ± 0.14 mm (mean ± st. dev.; measurement error of 82 μm); with a non-linear increase in force up to maximum of 2.0 kN.

The differences between the markers initial positions computed automatically and extracted manually were lower than 90 μm (i.e., about one pixel) in the transverse plane where bulge is computed. They were lower than 350 μm (i.e., about four pixels) in the axial direction whereas the axial distance between markers was about 800 μm.

**FIGURE 6:**
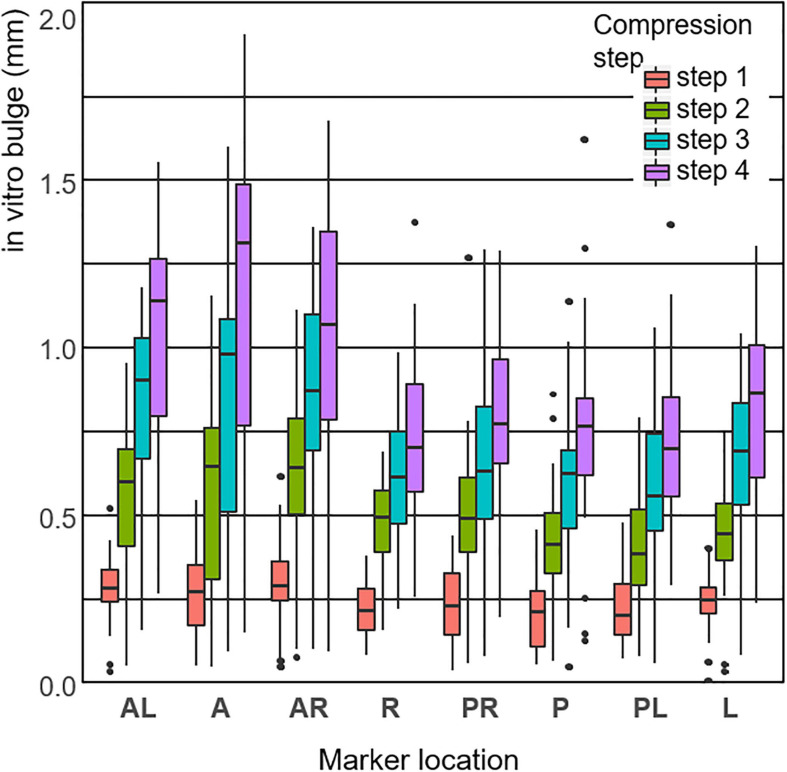
Box-plot distribution of the *in vitro* surface disc bulge, measured by marker location (defined in Figure 2) and load step.

The measured disc bulge across all steps and all markers was 0.24 ± 0.11 mm, 0.48 ± 0.22 mm, 0.67 ± 0.30 mm, and 0.84 ± 0.36 mm (mean ± st. dev. across all specimens and markers) for displacement steps 1–4, respectively (Figure 6). Generally, the anterior bulge was larger than the posterior bulge, with the differences being significant between some regions at each step (Table 2).

**TABLE 2:** Region pairs at which a significant difference in *in vitro* bulge is observed (A, anterior; P, posterior; L, left; R, right), comparison made for the bulge normalised with respect to the applied displacement (numbers indicate the compression steps at which the difference occurs).

Surface regions	L	PL	P	PR	R
**AL**		3, 4	3, 4		4
**A**	4	3, 4	3, 4	4	4
**AR**		2, 3, 4	1, 2, 3, 4		4

### Calibration of *in silico* Material Parameters Using *in vitro* Load-Displacement Data

The calibration of compressibility values performed for each specimen led to a convergence of the RMS difference in the force for five specimens (and all nucleus model types), while for the final one it terminated due to convergence of the values of the parameters. For this specimen, the achieved RMS difference in force for all nucleus model types was between 10 and 15% of the load experimentally measured at displacement step 3.

The optimisation yielded equivalent Poisson’s ratio values for the AF (mean ± std. dev.) of 0.494 ± 0.004 in the std. cyl models, and of 0.438 ± 0.08 for the other two nucleus model types (no significant differences between these two types). Equivalent Poisson’s ratios for the NP were 0.493 ± 0.003 in the std. cyl models, and 0.482 ± 0.01 for the other two nucleus model types.

**FIGURE 7:**
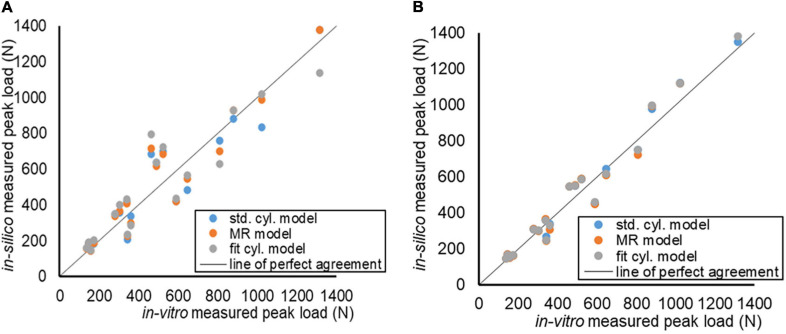
Agreement of the *in vitro* and *in silico* load measurements, for each specimen and each model type. **(A)** Models using 1-to-1 calibration of the compressibility value. **(B)** Models using the average compressibility value.

Following one-to-one calibration, the concordance correlation coefficients for loads at all time steps for all specimens were above 0.988 for all nucleus model types; while a reduction was observed for each of them in the average compressibility models (see Table 3 and Figure 7).

**TABLE 3:** Concordance coefficients (CCC with 95% confidence interval in brackets) across all specimens and all displacements steps of *in silico* load and markers bulge (disregarding markers within the lowest 10 percentile of experimental bulge).

	Load		Bulge	
Model type	1-to-1 calibration	Average compressibility	1-to-1 calibration	Average compressibility
Std. cyl. model	0.992 (0.985−0.998)	0.972 (0.910−0.979)	0.681 (0.485−0.735)	0.591 (0.385−0.721)
Fit. cyl. model	0.988 (0.964−0.996)	0.886 (0.820−0.972)	0.682 (0.429−0.791)	0.576 (0.424−0.672)
MR model	0.995 (0.961−0.999)	0.972 (0.831−0.989)	0.663 (0.406−0.813)	0.599 (0.524−0.636)

### Comparison of *in vitro* and *in silico* Values of Disc Bulge

**FIGURE 8:**
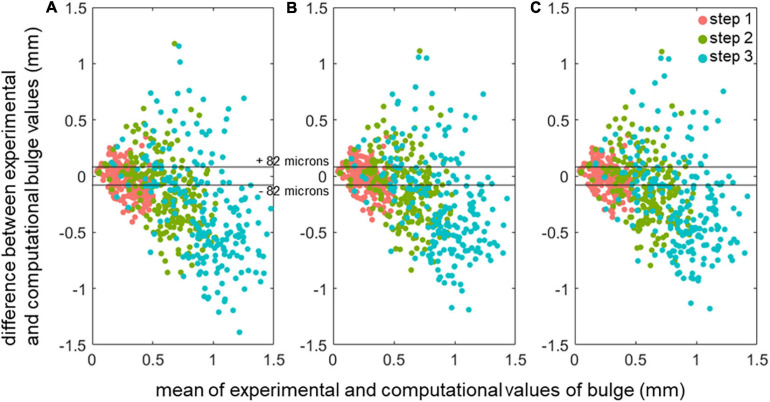
Comparison of the *in silico* predicted displacements of the disc marker points versus the measured *in vitro* values, shown as mean-difference plots for the three different model types with the 1-to-1 calibration. **(A)** std. cyl. models **(B)** fit. cyl. models **(C)** MR models. The image resolution band is shown with horizontal lines.

No differences were seen between nucleus model types in the ability to model bulge values (Table 3). Across all specimens, all model types and all marker locations, about 36% of bulge data had a difference between *in vitro* measurements and *in silico* measurements lower than the image resolution (82 μm). The remaining cases were evenly split between those where the *in silico* values were smaller than the *in vitro* values and those where they were higher, as shown in Figure 8 for the 1-to-1 calibration models (similar results are found for the average calibration model, data available at Mengoni and Wilcox, 2019). For all models, no significant differences were observed for the computational bulge values between marker locations, at any of the displacement steps.

## Discussion

The present work successfully developed a methodology to quantify the IVD bulge *in vitro*, enabling observation of lateral surface displacement of the IVD on CT images under axial load. The pre-loaded CT images and MRI data were used to create specimen-specific image-based FE models of the IVD, and then used to analyse the effect of different approaches to combining geometrical information from MR images with the CT data. While a systematic methodology was developed to replicate the behaviour of the discs just after loading, no clear advantage of using MR geometrical data was seen in this bovine tissue study. The FE models were designed to analyse the variability between samples tested in the lab, the direct-controlled approach used in this study (direct comparison of data measured *in vitro* with data computed *in silico* on the same specimen, and controlled loading scenario) provided confidence that the methodology can capture the variation between samples rather than a generic behaviour. However, it was not meant to model clinical scenarios, nor did it indicate validity in other loading scenarios or for different outputs of interest. Key discussion points presented are with respect to the intended context of use and should not be extrapolated to other contexts. In particular, the FE modelling framework presented is not able to replicate any information related to time-dependent behaviour, changing level of hydration, or local interaction between different tissue types.

### Experimental Characterisation of IVD Bulge

A number of methods of measuring disc bulge *in vitro* have been developed previously but have not yet been employed to make direct comparisons with specimen-specific FE models. The use of a high-resolution qCT in this study facilitated the capture of precision 3D morphology and provided the advantage of enabling the assignment of greyscale-dependent material properties to the hard tissues in the FE models. It was shown previously that capturing the bone behaviour in static models of osteodiscs is important to replicate the apparent stiffness of the specimen (Sikora et al., 2018). Although MRI is able to provide more information on the internal structural deformations, the 3D resolution and spatial accuracy would not have been sufficient for this study. Instead, fiducial markers were used on the external disc surface, which enabled a one-to-one comparison with corresponding nodal displacements on the FE model. This approach had the advantage in requiring no additional processing steps or assumptions to be made, meaning that the measurement error was due to the image resolution alone. Combined with an initial CT for greyscale-dependent properties of the bone, similar outcomes could be obtained by using 3D surface imaging rather than CT to compute the displacement of the fiducial markers, reducing the exposure of the tissue to repeated radiation and likely enabling shorter scanning times. Using an imaging method with shorter scanning times would enable measuring the bulge at several time points during relaxation, similarly to Heuer et al., 2007.

Regardless of the measurement method used, there are some limitations to the approach employed because of the time-dependent behaviour of the IVD tissue. In this study, the relaxed tissue was imaged, requiring the specimen to be held for 90 min prior to imaging in each step. The change in bulge during relaxation could not be estimated with the method used. While it has been shown in the canine thoracolumbar spine (Cassidy et al., 1990) that the bulge recovers at low compression levels (5% compression), it is less the case at slightly higher compression (∼15% compression), and there are no experimental data available at the higher compression levels applied in this work.

From the measurements made, the anterior region of the disc was seen to bulge more than the posterior. Similar results were obtained for human tissue under neutral axial compression, with larger radial displacements in the anterior than in the posterior regions on sagittal MRI slices (O’Connell et al., 2011). While this study used healthy bovine discs, the methodology employed could be applied to human discs, for example to examine the effects of different pathologies or herniation risks. In particular, the experimental and imaging framework developed here allows to capture some of the asymmetry in bulge which can also be observed clinically (Fardon et al., 2014). The MR imaging protocol used was optimised for human (cadaveric) spine imaging (Wijayathunga et al., 2019) and as such the framework is directly translatable to cadaveric tissue.

### *In silico* Agreement of *in vitro* Measurements – Load

In this work, compressibility values used for the finite element models were calibrated to minimise the force difference between the force recorded upon loading and the force required in a static FE model of the IVD. Previous work on similar *in vitro* and *in silico* models had shown that compressibility was a major factor influencing stiffness outputs (Mengoni et al., 2017). The converged equivalent Poisson’s ratios in this study were in the same range as found otherwise in the literature for IVD FE models that do not employ a multi-phasic representation of the disc (e.g., Marini and Ferguson, 2014; Adam et al., 2015; Casaroli et al., 2017), with almost incompressible materials. However, there was a significant difference in the outcome for the nucleus compressibility depending on how its volume was modelled: models with a large, standardised, nucleus required the use of a less compressible material model than a smaller sized nucleus, for which the size was based on MR image evaluation. Therefore, when using simple material models of the nucleus, the compressibility value should be chosen consistently with the manner in which the NP geometry is modelled.

The initial force was used for model calibration as it represents a more physiological value than the force recorded after a long relaxation without control of hydration level (on average 10 times lower). The reduction in the experimental force during relaxation is both due to the tissue relaxation itself and the change in hydration. The tissue compression leads to a reduction in water content, due to the fluid leaking out through the disc surface, which reduces the compressibility of the tissue. The exposure to air in itself has been observed to reduce the stiffness (Wilke et al., 1998). While not reported in this work, performing the same calibration for relaxed load values generated equivalent Poisson’s ratios which are not physically relevant (values average of 0.2 for the nucleus, and the annulus requiring an auxetic behaviour; data available at Mengoni and Wilcox, 2019). The bulge values were on the contrary measured experimentally only in a relaxed position. This discrepancy constitutes the main limitation of this study as the methodology does not allow to measure the instantaneous bulge or the change in bulge during relaxation. This method was based on the assumption that the disc bulge does not recover significantly during relaxation, with previous work on canine spine demonstrating reduced bulge recovery with compression levels of 15% and higher (Cassidy et al., 1990). For human tissue, the change in bulge during creep experiments has been observed as being negligeable with respect to most measurement resolution methods (Heuer et al., 2007).

With relatively simple material models for all tissues and testing conditions, the agreement between *in silico* and *in vitro* load magnitudes were better than previous studies (Maquer et al., 2015; Mengoni et al., 2017), irrespective of the way the NP geometry was represented and whether 1-to-1 calibration was performed, or average values were used. The main difference in the current work probably is due to the attention and effort taken with regard to the dissection of the soft tissues surrounding the IVD. In the present study, all soft tissues were carefully removed to leave a bare IVD surface only, enabling the outer surface of the disc to be easily and accurately segmented from CT images. Previous studies, where excess soft tissue had been retained, have had to use an artificial method to identify the outer disc surface (Sikora et al., 2018). This had a major effect on the ability to build FE models from micro-CT scans and indicated that being able to reconstruct the overall volume of the IVD is a major factor in being able to model accurately load data in compression. The tissue preparation effort here was well paid off by the improved agreement of the models.

While validation work would be required to know if the average compressibility values can be used on other specimens, the fact that the agreement was substantial both for 1-to-1 calibration and average values increased confidence on the validity of the average values for other specimens. Therefore, it may not be necessary to derive material properties through specimen-specific calibration, providing appropriate average values were already available.

It was found that, for bovine tissues models, using geometry information for the NP derived from MR images did not improve the ability of the models to predict load behaviour; this is likely due to the relatively arbitrary definition of tissue separation, even from MR data. Using MR sequences to define the outer AF boundary, or to give information about material properties, for example from T2 mapping, ρ_H_-weighted or DTI sequences (Reutlinger et al., 2014; Stadelmann et al., 2018; Chetoui et al., 2019), would possibly be better ways to add value from MR data, especially for degenerated discs or when time-dependency behaviour is of interest, for which the level of tissue hydration is important.

In addition, for two of the model types, the agreement between the *in silico* and *in vitro* load data did not vary greatly between cases where the material compressibility values were derived for each specimen separately and cases where average values were used. There was only an improvement in using direct calibration of each specimen, as opposed to average values, in the case where the NP was represented as a cylinder centred in the disc and with volume derived from MR data (fit. cyl. models).

### *In silico* Agreement of *in vitro* Measurements – Bulge

Including geometry information derived from MR images into the modelling methodology, whether only through evaluation of the nucleus volume or also by including its shape, did not produce significant differences in the accuracy of the *in silico* surface bulge of the IVD under compression. In particular, none of the nucleus model types were able to capture the differences between anterior and posterior bulge. It should be noted that the bovine disc is highly circular and thus that models in this study assumed a circular nucleus (except for the MR-models). This would not translate to the human intervertebral disc, and this conclusion may not apply in clinical scenarios. However, developing modelling methodologies valid for the bovine disc had interest in itself as the bovine spine is often used as a preclinical *in vitro* model for evaluating disc repairs. This work showed that simple *in silico* models of such preclinical evaluation are likely accurate enough to be used as complement to *in vitro* evaluation.

When evaluating the predicted disc bulge using the pointwise comparison of 40 locations on the disc surface, the level of agreement was only slightly improved when using models with a 1-to-1 calibration of compressibility properties versus those assigned an average value, and remained relatively poor for all nucleus geometry types. Accounting for the difference in lamellar strength and fibre orientation in the radial direction of the IVD (Elliott and Setton, 2000; Holzapfel et al., 2005) might enhance the ability to predict bulge. Differentiating the inner and outer annulus, with a core nucleus shape derived from MR and a more elliptical inner annulus, could also be used to the same aim. However, the large scatter seen in the mean-difference plot, without any clear locational trends, suggested these potential model improvements would not fully account for the current disparities. Errors in bulge may also be caused by the *in vitro* values being computed from images in a relaxed position while the *in silico* models were calibrated against peak force values.

Relatively simple constitutive models were used in this work and do not account for any fluid-flow effect of the tissues composing the IVD. This approach has shown to be valid when the purpose of the *in silico* models is to replicate global properties of osteodiscs or functional units, such as apparent stiffness (e.g., Mengoni et al., 2017; Sikora et al., 2018), facet joint contact (e.g., Ayturk and Puttlitz, 2011; Mengoni et al., 2016) or range of motions (e.g., Ayturk and Puttlitz, 2011; Sharabi et al., 2019). The present work showed that this modelling approach has some capacity to replicate more localised values such as disc bulge but the methodology was limited, including by the image acquisition time which required to measure bulge values after relaxation while the modelling interest was at peak load. It should be noted that changes in bulge under constant applied displacement are likely to be low, and only a single time-point was captured for each applied displacement. Therefore, the capacity to assess viscoelastic material models using the approach presented here is somewhat limited. Nevertheless, other material models could be partially assessed using the load relaxation data provided with this paper.

Despite the limitations of the current modelling method for predicting regional disc bulge, the combined *in vitro* and *in silico* methods presented in this study did provide an improved route for the evaluation of future modelling approaches, including those incorporating time-dependent material properties.

### Conclusion

The experimental arm of this study presented a new methodology for examining 3D IVD bulge under axial load. The methodology allows direct 1-to-1 comparison of disc surface displacement with corresponding FE models and has the potential to be used to examine the effects of tissue degeneration or different modes of loading.

On the computational aspects, this study suggests that, in order to obtain substantial agreement on load magnitude, significant care should be taken on reconstructing the external geometry of the IVD. The results from the different approaches to modelling the NP indicate that the use of an MRI-derived NP boundary does not improve capacity to capture bulge in a bovine model calibrated for load.

## Data Availability Statement

The datasets presented in this study can be found in online repositories. The names of the repository/repositories and accession number(s) can be found below: University of Leeds data repository: https://doi.org/10.5518/586.

## Ethics Statement

Ethical review and approval was not required for the animal study because this study uses animal tissue obtained from the food chain.

## Author Contributions

MM contributed to the design and conception of the work, data acquisition, analysis, and interpretation. FZ-C contributed to the design of the work, data acquisition, and analysis. VW contributed to the design of the work, data acquisition, and interpretation. RW contributed to the design and conception of the work, data analysis, and interpretation. All authors contributed to the drafting and critically reviewing the manuscript.

## Conflict of Interest

The authors declare that the research was conducted in the absence of any commercial or financial relationships that could be construed as a potential conflict of interest.
